# The comparison of decay rates of infectious SARS-CoV-2 and viral RNA in environmental waters and wastewater

**DOI:** 10.1016/j.scitotenv.2024.174379

**Published:** 2024-06-30

**Authors:** Asja Korajkic, Brian R. McMinn, Adin C. Pemberton, Julie Kelleher, Warish Ahmed

**Affiliations:** a Office of Research and Development, United States Environmental Protection Agency, 26 West Martin Luther King Drive, Cincinnati, OH 45268, United States; b CSIRO Land and Water, Ecosciences Precinct 41 Boggo Road, Qld 4102, Australia

**Keywords:** Infectious SARS-CoV-2, Viral RNA, Decay, Temperature, Wastewater, Ambient water

## Abstract

Understanding the decay characteristics of severe acute respiratory syndrome coronavirus 2 (SARS-CoV-2) in wastewater and ambient waters is important for multiple applications including assessment of risk of exposure associated with handling wastewater samples, public health risk associated with recreation in wastewater polluted ambient waters and better understanding and interpretation of wastewater-based epidemiology (WBE) results. We evaluated the decay rates of infectious SARS-CoV-2 and viral RNA in wastewater and ambient waters under temperature regimes representative of seasonal fluctuations. Infectious virus was seeded in autoclaved primary wastewater effluent, final dechlorinated wastewater effluent, lake water, and marine water at a final concentration of 6.26 ± 0.07 log_10_ plaque forming units per milliliter. Each suspension was incubated at either 4°, 25°, and 37 °C. Samples were initially collected on an hourly basis, then approximately every other day for 15 days. All samples were analyzed for infectious virus via a plaque assay using the Vero E6 cell line, and viral gene copy levels were quantified with the US CDC’s N1 and N2 reverse transcriptase quantitative polymerase chain reaction (RT-qPCR) assays. The infectious virus decayed significantly faster (*p* ≤ 0.0214) compared to viral RNA, which persisted for the duration of the study irrespective of the incubation conditions. The initial loss (within 15 min of seeding) as well as decay of infectious SARS-CoV-2 was significantly faster (*p* ≤ 0.0387) in primary treated wastewater compared to other water types, but viral RNA did not degrade appreciably in this matrix until day 15. Overall, temperature was the most important driver of decay, and after 24 h, no infectious SARS-CoV-2 was detected at 37 °C in any water type. Moreover, the CDC N2 gene assay target decayed significantly (*p* ≤ 0.0174) faster at elevated temperatures compared to CDC N1, which has important implications for RT-qPCR assay selection for WBE approach.

## GRAPHICAL ABSTRACT

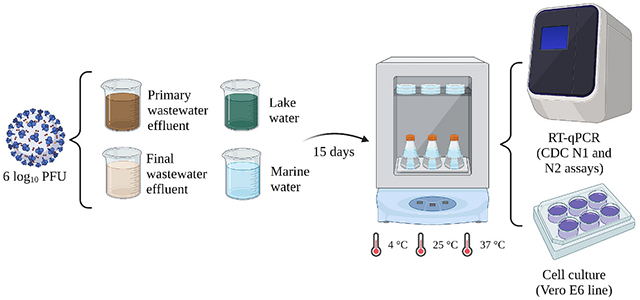

## 1. Introduction

The ongoing coronavirus disease 2019 (COVID-19) pandemic, caused by severe acute respiratory syndrome coronavirus 2 (SARS-CoV-2), an enveloped *Betacoronavirus* (Coronaviridae Study Group of the International Committee on Taxonomy of, V, 2020), has caused nearly 700 million cases and almost 7 million deaths worldwide as of Spring 2024 (Dong et al., 2020). SARS-CoV-2 is primarily transmitted through air via airborne transmission/inhalation of droplets and very small particles (Morawska and Cao, 2020; Yu et al., 2020), but virus is also excreted in feces (Wang et al., 2020; Xiao et al., 2020) and has been frequently detected in wastewater worldwide (recently reviewed in (Ahmed et al., 2022a; Ciannella et al., 2023; Dutta et al., 2022)). Because of this, wastewater surveillance programs have been deployed to better capture the infection rates in a community through wastewater-based epidemiology (WBE). Most research on SARS-CoV-2 detection in wastewater conducted to date has focused on quantification of viral RNA in and improvements in concentration and detection technologies. However, relatively little is known about persistence and decay of infectious SARS-CoV-2 and viral RNA in wastewater and surface waters, which can lead to uncertainties when estimating the risk associated with handling wastewater samples and public health risks from exposure to wastewater polluted ambient waters. Furthermore, gaining better understanding of SARS-CoV-2 decay in these matrices can aid in interpretation of WBE results.

The release of untreated or partially treated wastewater can contaminate surface waters via many different routes, including failing infrastructure and combined and sanitary sewer overflows. Wastewater contamination can pose a risk to human health through recreational activities, as well as possible ingestion if the same surface waters are used as a source for drinking water or for produce irrigation. The potential for contamination of surface waters by untreated or partially treated wastewater underscores the importance of understanding the persistence of SARS-CoV-2 in both water types. To date, there is limited information regarding decay characteristics of infectious SARS-CoV-2 due to the risks associated with propagating infectious virus, which requires the use of biosafety level 3 laboratories (BSL-3). Because of this, a majority of studies have instead focused on documenting decay characteristics of either the SARS-CoV-2 RNA (Ahmed et al., 2020a; Hokajarvi et al., 2021; Weidhaas et al., 2021), or surrogate viruses, such as murine hepatitis virus (MHV), transmissible gastroenteritis virus (TGEV) infecting pigs, feline infectious peritonitis virus (FIPV), human coronavirus 229E (HCoV229E), or Φ6 bacteriophage (Ahmed et al., 2020a; Aquino de Carvalho et al., 2017; Casanova et al., 2009; Casanova and Weaver, 2015; Gundy et al., 2009; Ye et al., 2016).

The work conducted on surrogate infectious viruses mainly focused on describing the effects of temperature, indigenous microbiota and particle association on decay. For example, in wastewater the decay of infectious Φ6 bacteriophage was shown to be significantly faster at higher temperatures (i.e., ≥ 25 °C) (Casanova and Weaver, 2015; Ye et al., 2016). A similar trend was also observed for infectious MHV and TGEV in wastewater and lake water (Casanova et al., 2009; Ye et al., 2016), and MHV RNA in wastewater (Ahmed et al., 2020a). Removal of indigenous microbiota, either through pasteurization or autoclaving, extended the persistence of infectious MHV and Φ6 bacteriophage, in river water (Aquino de Carvalho et al., 2017) and wastewater (Ye et al., 2016). Lastly, particle association has been demonstrated in wastewater for infectious MHV and Φ6 bacteriophage, and it has been suggested that it may be a mechanism by which other enveloped virus surrogates, such as human coronavirus 229E and FIPV, persist longer in wastewater (Gundy et al., 2009).

To date there are only a few studies that explored the assessment of infectivity through cell culture. For example, the decay of infectious virus was assessed in tap water, ambient water and raw wastewater at different temperatures or in the presence and absence of indigenous microbiota (de Oliveira et al., 2021; Lee et al., 2020; Sala-Comorera et al., 2021; Bivins et al., 2020). The stability of SARS-CoV-2 RNA was explored further, but mostly in the context of wastewater sample storage conditions and holding times (Hokajarvi et al., 2021; Weidhaas et al., 2021; Baldovin et al., 2021). Two additional studies compared persistence of SARS-CoV-2 RNA to that of surrogate viruses such as MHV and Pepper Mild Mottle Virus in raw wastewater and tap water, as well as wastewater settled solids at different temperatures (Ahmed et al., 2020a; Roldan-Hernandez et al., 2022).

In the current study, we expand on these findings by documenting the decay of infectious.

SARS-CoV-2 and the decline of viral gene copy (GC) levels over the course of 15 days in primary treated and final wastewater effluents, as well as lake and marine waters under three environmentally relevant temperatures, 4 °C, 25 °C and 37 °C.

## 2. Materials and methods

### 2.1. Wastewater and ambient water sample collection

A one-liter grab samples of primary treated wastewater and a one liter of final chlorinated wastewater effluent samples were collected from a local wastewater treatment plant, and two liters grab samples of ambient water were collected from Grayson Lake, KY (38°23′03” N and 83°01′34” W) by submerging the sterile collection bottles approximately 5 cm below the surface of the water. Samples were transported to the laboratory on ice and stored at 4 °C for 48 h. Prior to autoclave treatment (121 °C, 15 psi for 15 min per one liter) to inactivate indigenous microbial populations, final chlorinated wastewater sample was dechlorinated using anhydrous sodium thiosulfate (6.7 mg/L Sigma-Aldrich, St. Louis, MO), and a portion of Grayson Lake water was treated by adding sea salts (Sigma-Aldrich, St. Louis, MO) to a final target concentration of 35 ppt (*w/v*) to mimic typical ambient saltwater conditions. Furthermore, samples from both wastewater and ambient water types were inoculated onto Vero E6 cell line following protocol described in Subsection 2.5 to monitor for any potential infectious indigenous viruses or contaminants capable of producing cellular cytotoxicity. To confirm that no indigenous SARS-CoV-2 viral RNA was present in heat-sterilized samples, RT-qPCR testing was performed as described in Subsection 2.6.

### 2.2. Experimental microcosms

A series of microcosms was prepared to study the effects of water type (primary treated wastewater, final wastewater effluent, lake water, and salinity) and temperature (4°, 25° and 37 °C) on decay of infectious SARS-CoV-2 and viral RNA. A 100 mL aliquot of each water type was allowed to equilibrate at each of the three target temperatures for 30 min prior to addition of SARS-CoV-2 seed. A one mL volume of seeding suspension containing 6.26 ± 0.07 log_10_ PFU/mL was added to each water type and temperature combination and it was vortexed for ten min. The concentration of seeding suspension was selected to account for initial *v*/v dilution resulting from addition of one mL of viral suspension to 100 mL of each water type, and to allow for monitoring of infectious virus decay over time. Each seeded 100 mL sample was aliquoted into 30 mL triplicates, immediately followed by collecting one mL time zero (T_0h_) sample. Seeded samples (*n* = 36; 4 matrix types × 3 temperatures × triplicate for each) were incubated at three target temperatures and one mL samples were collected post incubation at the following timepoints: 1 h (T_1h_), 2 h (T_2h_), 4 h (T_4h_), 8 h (T_8h_), 24 h (T_24h_), 48 h (T_48h_), 96 h (T_96h_), 192 h (T_192h_), 264 h (T_264h_) and 360 h (T_360h_) in anticipation of persistence/decay of viral RNA. All samples were archived at −80 °C until further sample processing.

### 2.3. Vero E6 cell line propagation

Vero E6 cell line (Cat. #ATCC CRL 1586, American Type Culture Collection, Manassas, VA) was grown to 90–95 % confluency in 75 cm^2^, filter capped flasks (Thermo-Fisher Scientific, Waltham, MA) containing 30 mL of maintenance media consisting of Dulbecco’s Modified Eagles Medium (DMEM), with high Glucose with l-glutamine (Thermo-Fisher Scientific), and 10 % fetal bovine serum (FBS) (Thermo-Fisher Scientific). Flasks were incubated at 37 °C, under 5 % CO_2_ conditions.

### 2.4. Virus stock preparation

All experimentation involving infectious SARS-CoV-2, including virus stock preparation, microcosm set-up, infectious plaque assay and nucleic acid extractions, were carried out in a BSL-3 facility using standard BSL-3 practices and procedures which were approved by the Institutional Biosafety Committee of the University of Cincinnati (UC). One mL of infectious SARS-CoV-2 (Washington State strain, USA/WA1/2020), provided by Dr. Suman Pradhan (UC), was added to multiple 75 cm^2^ filter capped flasks containing 90–95 % confluent Vero E6 monolayer, and flasks were gently rocked every 15 min for one hour at 37 °C and under 5 % CO_2_ atmosphere to allow for infection to occur. Thirty mL of maintenance media was added, and incubation continued under the same conditions for 72 h. The flasks were then subjected to two freeze-thaw cycles at −80 °C, followed by centrifugation at 2500 × *g* for 30 min to pellet cellular debris. The resultant supernatant was syringe filtered (0.22 μm pore size), and virus stocks were frozen at −80 °C until use. Prior to experimental seeding procedures virus stock was titered by performing decimal dilution series in maintenance media, followed by infectious plaque assay and RT-qPCR.

### 2.5. Infectious plaque assay

A viral plaque assay was performed according to the previously published alternate protocol (Mendoza et al., 2020) for quantification of infectious SARS-CoV-2. Briefly, six-well plates (Corning, Corning, NY) were seeded with 4 × 10^5^ Vero E6 cells per well and incubated at 37 °C and at 5 % CO_2_ for three days to allow formation of 90 % confluent cell monolayer in each well. Prior to infection, media was aspirated, and the cell monolayer was washed with Dulbecco’s Phosphate Buffer Saline (DPBS) (Thermo-Fisher Scientific). In the early stages of the experiment, and for quantification of viral seed, serial dilutions of samples were prepared using infectious media as diluent. At the later time points, as infectious virus concentrations declined, samples were processed undiluted. Vero E6 cell monolayers were infected by adding 100 μL of the sample, followed by one hour incubation at 37 °C under 5 % CO_2_ atmosphere while rocking every 15 min. After the infection was completed, three mL of liquid overlay medium (L-OM; consisting of equal volumes of 3 % carboxymethylcellulose and overlay diluent (Mendoza et al., 2020), Thermo-Fisher Scientific) was added to each well, followed by a 72 h incubation at 37 °C under 5 % CO_2_ atmosphere. Next, the L-OM was aspirated, and the cell monolayer was washed with DPBS and fixed with one mL of 4 % paraformaldehyde (Thermo-Fisher Scientific) for one hour at room temperature. After one hour, fixative was aspirated, and each well was stained with 200 μL of 0.5 % crystal violet solution (Thermo-Fisher Scientific) for 15 min at room temperature. The staining solution was washed three times with one mL of sterile deionized water and plaque forming units (PFU) were enumerated. With each iteration of the viral plaque assay, three negative control wells were included (100 μL of maintenance media in lieu of sample).

### 2.6. RT-qPCR

All RT-qPCR experiments were conducted in accordance with applicable Minimum Information for Publication of Quantitative Real-Time PCR Experiments guidelines (MIQE) (Bustin, 2010) and The Environmental Microbiology Minimum Information (EMMI) guidelines (Borchardt et al., 2021). Viral nucleic acids were extracted from 200 μL portions of each sample using a Qiagen AllPrep^®^ PowerViral^®^ DNA/RNA (Qiagen, Valencia, CA) kit according to manufacturer instructions. One nucleic acid extraction blank (NAEB) was included with each extraction batch to monitor for potential extraneous contamination. Viral RNA (genomic copies [GC]) was amplified using the RNA Ultra-Sense^™^ One-Step Quantitative RT-PCR System (Applied Biosystems, Foster City, CA) within 24 h of nucleic acid extraction following manufacturer instructions. Briefly, each reaction consisted of 1.25 μL enzyme mix, 5 μL 5× reaction mix, 2.5 μL bovine serum albumin (at 0.2 mg/mL), 0.05 μL ROX reference dye, 11.2 μL nuclease free water, 2 μL template and 3.0 μL of 2019-nCoV Research Use Only (RUO) qPCR kit (Integrated DNA Technologies, Coralville, IA) containing pre-mixed primers and probe for CDC’s N1 and N2 assays (Centers for Disease Control and Prevention, 2020) for a total reaction volume of 25 μL. The RT-qPCR conditions were as follows: 1) holding stage 1 at 50 °C for 15 min, 2) holding stage 2 at 95 °C for 2 min, followed by 3) 40 cycles at 95 °C for 5 s and 60° for 30 s. All RT-qPCR reactions were performed in triplicate. and amplifications were carried out using QuantStudio 3 real-time qPCR system (Applied Biosystems) with the threshold manually set to 0.03. Six no template controls (NTCs) were included with each instrument run. Utilizing a previously described approach (Cao et al., 2012), potential inhibition for each assay was assessed by performing 10-fold dilution of each sample in sterile AE buffer (Qiagen) and comparing the resulting Cq values to those of undiluted samples. Under ideal conditions (i.e., assuming 100 % amplification efficiency), 10-fold dilution is expected to delay detection of the target by 3.32 cycles. Allowing for natural variability among the individual RT-qPCR replicates, the sample was considered inhibited if the diluted sample was one cycle less than expected following the measurement of the undiluted sample.

### 2.7. RT-qPCR standard curve

Armored RNA Quant^®^ SARS-CoV-2 provided at 1 × 10^11^ copies per mL by the manufacturer (Asuragen, Austin, TX) was used as a reference RNA standard and was extracted and quantified as described above. The most probable number approach (McMinn et al., 2016) utilizing ten decimal dilution series (ten replicates each) was applied to determine the number of gene copies for each assay remaining after the extraction procedure. Based on these concentrations, serial dilutions spanning 10–10^6^ GC per 2 μL were prepared in sterile AE buffer for calibration standards. A standard master curve based on five individual standard curves for each assay was prepared and used for quantification of all samples in this study. Lower limit of quantification (LLOQ) was defined as the 95 % prediction upper limit of 10 copy RNA standard dilution (Shanks et al., 2016).

### 2.8. Data analyses

All concentrations were log_10_ transformed prior to data analyses. The decay of SARS-CoV-2 was calculated as cumulative log_10_ reduction (log_10_ C_0_ − log_10_ C_T_) where C_T_ represents concentration at different sampling time points (T_1h_, T_2h_, T_4h_, T_8h_, T_24h_, T_48h_, T_96h_, T_192h_, T_264h_ and T_360h_) and T_0_ represents the starting concentrations (Tables S1-S3). We opted to use log_10_ reduction approach over the first order decay rate constants as the latter can cause inconsistencies in decay rate estimates since microbial decay is rarely linear and typically displays bi-phasic curve (Brooks and Field, 2016). In order to facilitate potential future comparisons with studies that utilized first order decay rate constants we have provided raw data in Supplementary Materials (Tables S4-S6). GraphPad Prism version 8.1.2 (GraphPad Software, La Jolla, CA) was used to conduct all statistical analyses. A two-way analysis of variance (ANOVA) (Table 2) with Tukey’s multiple comparison tests (Tables 3 and 4) was used to evaluate the effect of two factors (temperature and water type), as well as any interaction between them, on SARS-CoV-2 decay. An ordinary one-way ANOVA with Tukey’s multiple comparison test was employed to compare the initial loss of viral signal (the difference in concentrations between viral seed and T_0h_ samples) and difference in decay between infectious SARS-CoV-2 and viral RNA. Paired *t*-tests were used to assess difference in decay of N1 and N2 RT-qPCR signals. All statistical analyses were performed at *α* = 0.05.

## 3. Results

### 3.1. RT-qPCR performance metrics, inhibition assessment and QA/QC outcomes

RT-qPCR performance metrics (i.e., slope, amplification efficiency, R^2^, y-intercept and LLOQ) for N1 and N2 assays were within recommended MIQE guidelines (Bustin et al., 2009) and are summarized in Table 1. Amplification inhibition was not detected in any of the samples for either assay. All 120 NAEB and 480 NTC reactions were below the LLOQ of individual assays, indicating an absence of external RNA contamination. The cell culture monitoring for potential infectious indigenous viruses and cytotoxicity, as well as RT-qPCR testing for the indigenous SARS-CoV-2 RNA in water and wastewater samples indicated none was present. Furthermore, no PFUs were detected in the negative cell culture controls for the duration of the study, indicating absence of external contamination.

### 3.2. Initial loss

We had observed considerable initial loss of infectious virus signal which occurred within 15 min of SARS-CoV-2 addition to different water types (Table S4). The 15-min interval refers to the time elapsed prior to collection of T_0h_ samples. The initial loss of infectious SARS-CoV-2 ranged from 0.65 to 1.60 log_10_ PFU, and it was significantly higher in primary effluent (*p* ≤ 0.0157) compared to other water types and also significantly higher (*p* ≤ 0.0473) than the loss of viral RNA (negligible – 0.89 log_10_), in all the water types (Tables S5, S6). Additionally, there was no significant difference in initial loss between N1 and N2 assays.

### 3.3. Decay of infectious SARS-CoV-2 and viral RNA

**Fig. 1.**
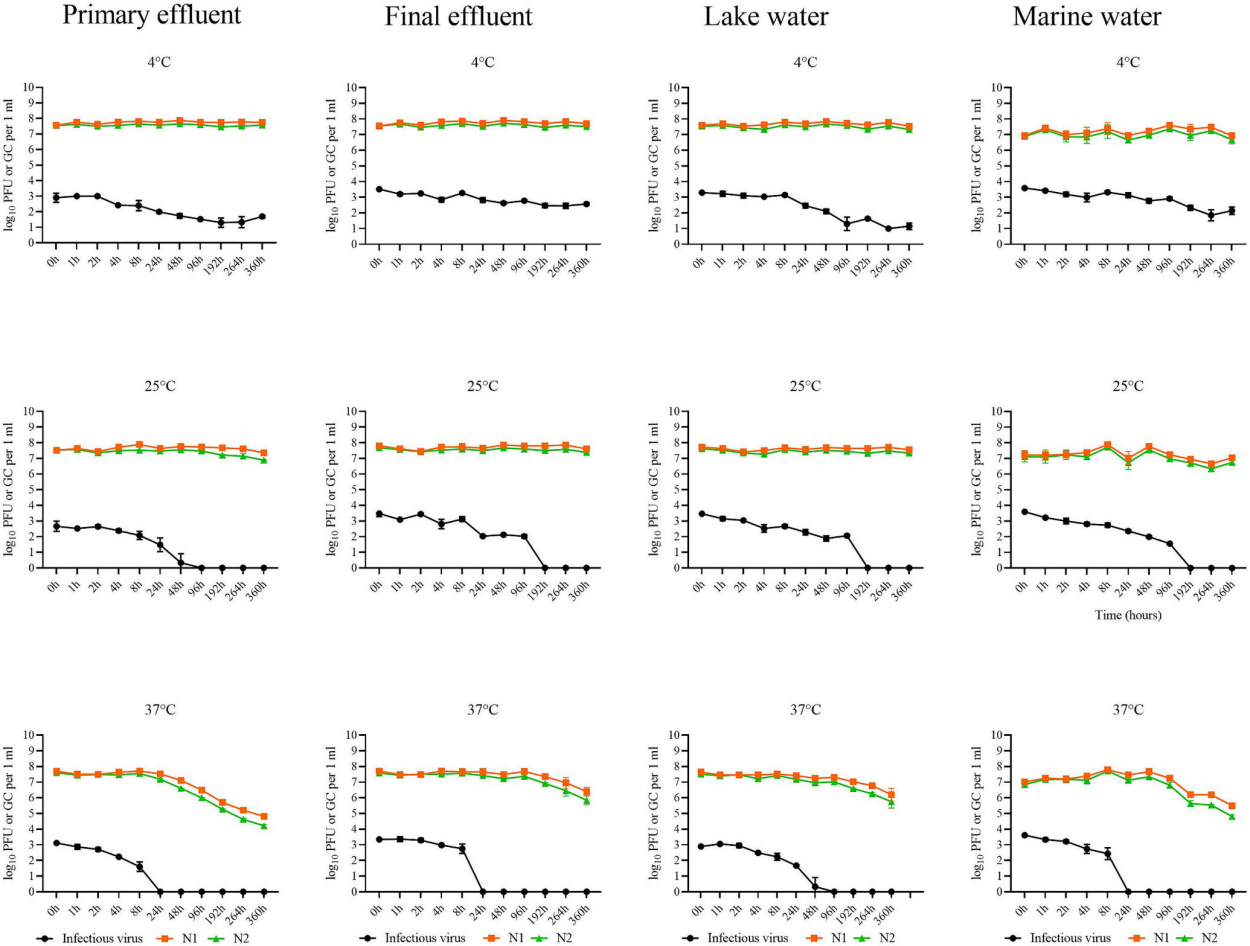
Change in concentrations of infectious SARS-CoV-2 (log_10_ PFU per mL) and viral RNA (log_10_ GC per mL) in primary treated wastewater, final wastewater effluent, lake water and marine water over time (T_0h_-T_360h_) and under different temperature regimes (4 °C, 25 °C, 37 °C). Error bars represent standard deviation across three replicate samples.

For the duration of the study, and irrespective of the treatment, average concentrations of infectious SARS-CoV-2 were significantly lower (*p* < 0.0001) compared to viral RNA by 4.94 log_10_ and 4.78 GC log_10_ for N1 and N2 assays (Fig. 1) respectively. The decay of viral RNA was much slower compared to infectious virus and it varied across temperature and water type combinations. For example, infectious virus remained detectable in all the water types at 4 °C through T_360h_, but at 25 °C the signal was lost at either T_48h_ (primary treated wastewater) or T_96h_ (remaining water types) (Fig. 1). Additionally, at 37 °C, it persisted until T_24h_ in lake water, but only until T_8h_ for the other water types (Fig. 1). This disparate pattern precluded comparisons of decay characteristics of infectious SARS-CoV-2 and viral RNA for all time points and treatment combinations. Therefore, we opted to do the comparisons for the last time point at each temperature when infectious virus was detected in all four water types. Specifically, this included decay at T_360h_ (4 °C), T_48h_ (25 °C) and T_8h_ (37 °C). At each of these time points and in all four water types, infectious virus decayed significantly faster compared to viral RNA (*p* ≤ 0.0214).

Finally, because viral RNA remained detectable for the duration of the study, we were able to compare the decay characteristics of N1 and N2 assays at T_360h_ at all three temperatures and in all four water types. At 4 °C, there was no statistically significant difference in decay in any water type (*p* ≥ 0.1487), but at 25 °C N2 decayed significantly faster than N1 in primary and final wastewater effluent, as well as marine water (*p* ≤ 0.0174) (Fig. 1). This trend was even more apparent at 37 °C, where N2 decayed significantly faster across all four water types (*p* ≤ 0.0092) (Fig. 1).

### 3.4. Effect of temperature and matrix on infectious SARS-CoV-2 decay

Due to uneven decay of infectious SARS-CoV-2 across different temperatures and water types, the effect of treatment variables was assessed at T_8h_ for all treatment combinations and at T_48h_ for all four water types, but for 4 °C and 25 °C only (Tables 2-4). The effect of all treatment variables was evaluated for N1 and N2 assays at T_8h_, T_48h_ and T_360h_ (Tables 2-4).

The temperature had a strong effect on decay of infectious SARS-CoV-2 for the duration of the study (Fig. 1). At T_8h_, temperature was a significant factor (*p* < 0.0001), contributing 40 % to the observed variability in the dataset (Table 2). Within this timeframe, infectious virus decay at 37 °C was significantly faster compared to lower temperatures (*p* ≤ 0.0234) (Table 3), and this was especially evident in the primary wastewater effluent (Fig. 1, Table 3). The trend of significantly (*p* < 0.0001) accelerated decay due to higher temperatures continued at T_48h_, affecting variability by nearly 56 % (Table 2). At this time point, no infectious viruses persisted at 37 °C in any of the water types, and although the decay was significantly (*p* ≤ 0.0388) faster at 25 °C compared to 4 °C in final wastewater effluent and marine water, there was no significant difference for the lake water (Table 3).

Water type significantly (*p* ≤ 0.0071) influenced infectious SARS-CoV-2 decay at T_8h_ and T_48h_, but the effect was not as pronounced as the effect of temperature (Table 2). At T_8h_, water type was a relatively minor contributor (~17 %), and this increased slightly to ~26 % at T_48h_ (Table 2). Specifically, at T_8h_ and at 37 °C only, infectious SARS-CoV-2 decayed significantly (*p* ≤ 0.0387) faster in primary wastewater effluent compared to final wastewater effluent and lake water (Table 4). The pattern of significantly (*p* ≤ 0.0007) greater reduction in primary wastewater effluent was more evident at T_48h_ (Table 4), as infectious SARS-CoV-2 decayed faster in this water type compared to the remaining three. However, this was evident only at 25 °C, as there were no statistically significant differences in decay by the water type at 4 °C. It is important to note that the interaction of variables was also significant (*p* = 0.0027) at T_48h_ (Table 2), suggesting that the observed water type effect was likely temperature dependent.

### 3.5. Effect of temperature and matrix on viral RNA decay

Unlike infectious SARS-CoV-2, temperature was not a significant contributor to decay of viral RNA prior to T_48h_, when it accounted for ~25 % of variability (Table 2). At this time point, decay was significantly faster (*p* ≤ 0.0137) at 37 °C compared to 4 °C, in primary and final wastewater effluent and lake water, but not marine water (Table 3). There were no significant differences in decay between 4 °C and 25 °C, or between 25 °C and 37 °C regardless of the water type. At T_360h_, the effect of temperature remained significant (*p* < 0.0001), and its contribution to the observed variability in log_10_ reductions increased to ~80 % (Table 2). At this time point, the decay was significantly (*p* < 0.0001) faster at 37 °C compared to both lower temperatures, irrespective of the water type (Table 3).

Unlike the temperature, water type was a significant contributor (*p* < 0.0001) to variability in the dataset (~60 %) at T_8h_ (Table 2) at 25 °C and 37 °C, but not 4 °C (Table 4). At this time point (T_8h_), viral RNA generally degraded less in marine water compared to the other water types, but there were some distinct differences in decay patterns between N1 and N2 targets. For example, N1 decay was significantly (*p* ≤ 0.002) faster (Table 4) in lake water and final wastewater effluents (compared to marine water) at 25 °C and 37 °C, but there were no other statistically significant differences. N2, on the other hand, persisted significantly (*p* ≤ 0.013) more (Table 4) in marine water compared to the remaining three water types at 25 °C, while at 37 °C, decay was faster (*p* = 0.0001) in primary effluent compared to marine waters. At T_48h_, water type remained significant (*p* < 0.0001) contributor to decay, but its effect was reduced to ~33 % (Table 2) and the general trend of extended persistence in marine waters at 25 °C and 37 °C continued (Table 4). For example, N1 decayed significantly less (*p* ≤ 0.0045) in marine waters compared to final wastewater effluent (25 °C, 37 °C), lake water (25 °C and 37 °C) and primary wastewater effluent (37 °C) (Table 4). The effect of water type on N2 decay was more pronounced, as it persisted significantly (*p* ≤ 0.0152) more in marine water compared to lake water (25 °C and 37 °C), final and primary wastewater effluent (37 °C), and in final vs primary wastewater effluent (37 °C) (Table 4). At T_360h_, water type remained significant factor (*p* < 0.0001) at 37 °C, but its influence diminished even further (~5 %) (Table 2) with notable shifts in persistence patterns across different water types. Namely, at this time point, decay of both N1 and N2 was significantly faster in primary wastewater effluent compared to all the other water types (*p* < 0.0001). Finally, interaction of variables was a significant (*p* ≤ 0.0271) contributor to the observed variability in the dataset for T_8_, T_48_ and T_360_ (Table 2), indicating that the effect of temperature and water type are inter-dependent.

## 4. Discussion

Since the onset of the COVID-19 pandemic, researchers worldwide have published unprecedented numbers of manuscripts detailing various aspects of SARS-CoV-2. A simple search for the keyword “SARS-CoV-2” of in one article repository database (i.e., https://pubmed.ncbi.nlm.nih.gov/) yielded over 220,000 papers, all generated in a little over three years. In the field of environmental microbiology, the primary research focus has been on wastewater-based surveillance efforts and methodological improvements, while only a minor subset of studies explored decay characteristics of infectious SARS-CoV-2 (de Oliveira et al., 2021; Lee et al., 2020; Sala-Comorera et al., 2021; Bivins et al., 2020). Even though the risk of transmission of infectious SARS-CoV-2 through environmental waters is low (Ransome et al., 2023; Sobsey, 2022; Wang et al., 2022), the World Health Organization issued the report (World Health Organization, 2020) highlighting the need for research exploring persistence of SARS-CoV-2 viral particles in environmental waters and wastewater. We aim to expand on these works by comparing decay characteristics of infectious SARS-CoV-2 and its RNA in different types of environmental waters and wastewater, under temperature regimes reflective of seasonal temperature fluctuations.

Overall accelerated decay of infectious virus was observed compared to that of viral RNA, irrespective of the temperature or water type. Additionally, we also noted a large discrepancy between concentrations of infectious virions compared to RNA GC (nearly 5 log_10_ difference) throughout the study (McMinn et al., 2023). While these ratios of RNA to infectious virions is not unusual (i.e., multiple target GC per virion or defective viral particles incapable of infecting the host cell), the difference is typically less dramatic (i.e., 1–2 log_10_) (Dovas et al., 2010; Gallagher and Margolin, 2007; Iwanami et al., 2017). The greater difference that we observed could be attributed to an abundance of subgenomic RNA (sgRNA) in the nucleocapsid protein region of SARS-CoV-2, a part of the viral genome that that generates the most sgRNA species and represents all the sgRNA expressed by the virus (Kim et al., 2020). The sgRNA intermediary is co-detected by CDC’s N1 and N2 assays, along with the genomic RNA (Kim et al., 2020; Lu et al., 2020), and this could also be at least one of the reasons for unsuccessful infectious SARS-CoV-2 cultivation attempts from wastewaters that had quantifiable viral RNA (McMinn et al., 2023; Rimoldi et al., 2020).

A considerable initial loss (within 10–15 min of being introduced into each water type) of SARS-CoV-2 occurred, which was more pronounced for infectious virions than the viral RNA, and it was more severe in primary treated wastewater compared to other water types. This finding is in line with previous reports for SARS-CoV-2, HCoV 229E and FIPV (Gundy et al., 2009; McMinn et al., 2023), and it could be attributed to either irreversible absorption to wastewater particulates or viral envelope degradation by chemicals present in wastewater such as surfactants, solvents and pharmaceuticals (Gundy et al., 2009; Hart and Halden, 2020). Although less pronounced, the initial loss in other matrices could be attributed to the activity of extracellular enzymes, and adsorption to organic and particulate matter (Patel et al., 2021).

We observed faster decay of infectious SARS-CoV-2 in primary wastewater effluent compared to other water types at all temperatures except 4 °C throughout the study. This has been previously described only in comparison to decay in river water (de Oliveira et al., 2021), whereas our study investigated additional water types (lake water, marine water, or final wastewater effluents). While we observed a general trend of faster decay in marine waters compared to freshwater, it was not as notable as previously described (Lee et al., 2020; Sala-Comorera et al., 2021), possibly due to artificial marine conditions in the present study. This implies that other marine water characteristics in addition to salinity, such as alkaline pH, may contribute to faster decay in this water type. In fact, significantly faster decay at higher pH as compared to neutral conditions has been reported for other coronaviruses including HCoV 229E, the MHV and TGEV (Geller et al., 2012).

The dynamics of viral RNA decay in response to exposure to various water types were different compared to infectious SARS-CoV-2. For example, incubation in primary treated wastewater compared to other water types was not a factor in decay until later stages of the experiment (i.e., T_360h_). This finding supports the application of WBE for wastewater surveillance of SARS-CoV-2 and potentially other emerging viruses across different sampling strategies (e.g., time composited wastewater samples) as viral RNA remained detectable for extended periods of time. Furthermore, viral RNA was remarkably stable in the marine water with negligible degradation over the course of a 15-day incubation, a finding that has not been described before for SARS-CoV-2. Higher salt concentration likely facilitated formation of salt-bridges, which in turn stabilized RNA through electrostatic interactions (Levintov and Vashisth, 2021; Tarus et al., 2012; Stone et al., 2019). This finding implies that the adsorption-extraction concentration methods relying on addition of MgCl_2_ (Ahmed et al., 2020b), not only promote adsorption of enveloped viruses to membrane filter but may also protect the viral RNA from degradation. Consequently, addition of salts may be a viable option to stabilize viral RNA during extended holding and storage times.

Temperature had a profound effect on viral decay. This was especially evident with infectious virions, as the infectious signal persisted for 2 weeks at 4 °C but were largely unculturable after 8 h at 37 °C. Rapid decay (~8 h to ~48 h) of infectious SARS-CoV-2 at higher temperatures ≥20 °C has been reported by others for freshwater (de Oliveira et al., 2021; Lee et al., 2020; Sala-Comorera et al., 2021), marine waters (Lee et al., 2020; Sala-Comorera et al., 2021) and raw wastewater (de Oliveira et al., 2021; Bivins et al., 2020). Temperature dependent decay of infectious SARS-CoV-2 in final wastewater is novel, and our results indicate similar decay patterns to other water types where infectious virions persisted for 8 h at 37 °C, 96 h at 25 °C and for at least 360 h at 4 °C.

Even though viral RNA decay was negligible compared to decay of infectious viruses, we observed greater decay at higher temperatures, but the effect of temperature was not as pronounced compared to infectious virions. Accelerated decay of SARS-CoV-2 RNA at higher temperatures (≥ 25 °C) has been documented in wastewater and ambient waters previously (Ahmed et al., 2020a; Weidhaas et al., 2021; Lee et al., 2020; Sala-Comorera et al., 2021; Bivins et al., 2020; Roldan-Hernandez et al., 2022; Yang et al., 2022). However, unlike these studies, we observed greater sensitivity of N2 assay gene target to elevated temperatures, compared to N1. The assays target two distinct regions of nucleocapsid protein gene, and while it was outside of the scope of this study to ascertain the precise mechanism, our results imply that the portion of the gene targeted by the N1 assay is more stable. This finding may explain generally lower concentrations of SARS-CoV-2 RNA in the wastewater observed when using N2 assay compared to N1 (McMinn et al., 2023; Ahmed et al., 2022b; Kaya et al., 2022) and has important implications for the assay selection for WBE. Furthermore, an earlier review article indicated varied SARS-CoV-2 RT-qPCR performance metrics across several commonly used assays (Ahmed et al., 2022a). Combined with the dissimilar decay characteristics between the N1 and N2 assays, this may affect sensitivity of SARS-CoV-2 detection, especially in time-composited wastewater samples during summer months, or in (sub)tropical regions.

This study adds to the limited pool of knowledge regarding decay characteristics of infectious SARS-CoV-2 and its RNA. We have confirmed temperature sensitivity of infectious virions, as well as extended persistence of viral RNA observed by others. We also demonstrated extended persistence of viral RNA in comparison with the infectious virus, as well as considerable initial inactivation of infectious SARS-CoV-2 especially in primary treated wastewater effluent. Furthermore, this was the first report of greater sensitivity of N2 assay gene target to elevated temperatures compared to N1, which has important implications for the RT-qPCR assay selection for WBE applications. Finally, due to various logistical constraints associated with conducting experiments under BSL-3 laboratory conditions, it is important to note that the study was conducted under artificial conditions (i.e., in dark and in autoclaved water) and on a particular SARS-CoV-2 strain. Therefore, the conditions were likely conducive to the extended persistence, and caution should be exercised when extrapolating the findings to true ambient conditions and other SARS-CoV-2 variants.

## Supplementary Material

Supplement1

## Figures and Tables

**Table 1:** Summary of RT-qPCR performance metrics for N1 and N2 assays.

Parameter	N1	N2
Slope (average ± standard deviation)	−3.328 ± 0.060	−3.230 ± 0.252
R^2^ (average ± standard deviation)	0.998 ± 0.001	0.994 ± 0.010
Y-intercept range	35.836–37.525	33.802–36.897
AE^a^	103 ± 0.001	103.1 ± 0.003
LLOQ^b^ range	33.023–34.412	32.168–34.344

a Amplification efficiency (AE = 10^(−1/slope)^ − 1).

b Lower limit of quantification Cq value.

**Table 2:** Effect of treatment variables (temperature and water type) on SARS-CoV-2 decay. Statistically significant values are bolded.

Measurement	Time point (h)	Factor					
		Temperature		Water type		Interaction^a^	
		%^b^	*p* value	%^b^	*p* value	%^b^	*p* value
Infectious virus	T_8h_	**40.01**	**<0.0001**	**16.96**	**0.0071**	16.45	0.0524
	T_48h_^c^	**55.69**	**<0.0001**	**26.25**	**<0.0001**	**10.43**	**0.0027**
N1	T_8h_	2.612	0.2318	**57.32**	**<0.0001**	**19.91**	**0.0069**
	T_48h_	**25.06**	**<0.0001**	**34.70**	**<0.0001**	**31.63**	**<0.0001**
	T_360h_	**79.88**	**<0.0001**	**4.292**	**0.0003**	**12.16**	**<0.0001**
N2	T_8h_	0.658	0.6924	**62.64**	**<0.0001**	**15.54**	**0.0271**
	T_48h_	**26.71**	**<0.0001**	**32.18**	**<0.0001**	**31.79**	**<0.0001**
	T_360h_	**81.67**	**<0.0001**	**6.005**	**<0.0001**	**9.248**	**<0.0001**

a Interaction between treatment variables (temperature and matrix).

b Percent contribution of each treatment variable (temperature and matrix) to the observed variability in the dataset.

c Two-way ANOVA performed at only two temperatures (i.e., 4 °C and 25 °C) due to loss of infectious signal after 24 h in primary and final wastewater, as well as marine water.

**Table 3:** Tukey’s multiple comparisons test (effect of temperature). Only statistically significant comparisons at the last date analytes were detected in all treatment combinations are shown. Please see Tables S7, S8 for complete dataset.

Analyte	Time point	Water type^a^	Comparison	Average log_10_	reduction and standard deviation for first and second temperature	*P* value
Infectious virus	T_8h_	P	4 °C vs 37 °C	0.51 ± 0.58	1.52 ± 0.27	0.0084
			25 °C vs 37 °C	0.59 ± 0.27	1.52 ± 0.27	0.0189
		M	4 °C vs 37 °C	0.27 ± 0.10	1.18 ± 0.39	0.0234
N1	T_360h_	P	4 °C vs 37 °C	−0.18 ± 0.04	2.88 ± 0.17	<0.0001
			25 °C vs 37 °C	0.16 ± 0.10	2.88 ± 0.17	<0.0001
		F	4 °C vs 37 °C	−0.15 ± 0.07	1.31 ± 0.28	<0.0001
			25 °C vs 37 °C	0.20 ± 0.16	1.31 ± 0.28	<0.0001
		L	4 °C vs 37 °C	0.06 ± 0.04	1.43 ± 0.41	<0.0001
			25 °C vs 37 °C	0.16 ± 0.09	1.43 ± 0.41	<0.0001
		M	4 °C vs 37 °C	0.02 ± 0.25	1.52 ± 0.24	<0.0001
			25 °C vs 37 °C	0.16 ± 0.32	1.52 ± 0.24	<0.0001
N2	T_360h_	P	4 °C vs 25 °C	−0.02 ± 0.05	0.00 ± 0.02	0.0423
			4 °C vs 37 °C	−0.02 ± 0.05	3.39 ± 0.14	<0.0001
			25 °C vs 37 °C	0.65 ± 0.08	3.39 ± 0.14	<0.0001
		F	4 °C vs 37 °C	0.05 ± 0.10	1.74 ± 0.33	<0.0001
			25 °C vs 37 °C	0.29 ± 0.11	1.74 ± 0.33	<0.0001
		L	4 °C vs 37 °C	0.22 ± 0.08	1.76 ± 0.39	<0.0001
			25 °C vs 37 °C	0.28 ± 0.04	1.76 ± 0.39	<0.0001
		M	4 °C vs 37 °C	0.21 ± 0.30	2.05 ± 0.25	<0.0001
			25 °C vs 37 °C	0.36 ± 0.36	2.05 ± 0.25	<0.0001

a P (primaiy wastewater effluent), F (final wastewater effluent), L (lake water), M (marine water).

**Table 4:** Tukey’s multiple comparisons test (effect of water type). Only statistically significant comparisons at the last date analytes were detected in all treatment combinations are shown. Please see Tables S7 and S8 for complete dataset.

Analyte	Time point	Temperature	Comparison^a^	Average log_10_	reduction and standard deviation for first and second matrix	P value
Infectious virus	T_8h_	37 °C	P vs F	1.52 ± 0.27	0.59 ± 0.15	0.0203
			P vs L	1.52 ± 0.27	0.66 ± 0.30	0.0387
N1	T_360h_	37 °C	P vs F	2.88 ± 0.17	1.31 ± 0.28	<0.0001
			P vs L	2.88 ± 0.17	1.43 ± 0.41	<0.0001
			P vs M	2.88 ± 0.17	1.52 ± 0.24	<0.0001
N2	T_360h_	37 °C	P vs F	3.39 ± 0.14	1.74 ± 0.33	<0.0001
			P vs L	3.39 ± 0.14	1.76 ± 0.39	<0.0001
			P vs M	3.39 ± 0.14	2.05 ± 0.25	<0.0001

a P (primary wastewater effluent), F (final wastewater effluent), L (lake water), M (marine water).

## Data Availability

Data will be made available on request.

## HIGHLIGHTS

- Infectious SARS-CoV-2 and viral RNA decay in different water types/temperatures.
- Infectious SARS-CoV-2 decayed faster than viral RNA in all treatment combinations.
- Temperature was important driver of decay for all analytes.
- Impact of water type (ambient, wastewater) was not as pronounced as temperature.
- N2 gene assay target decayed faster than N1 gene target at higher temperatures
