# Supplementary material for: The comparison of decay rates of infectious SARS-CoV-2 and viral RNA in environmental waters and wastewater
Source: Sci Total Environ. Author manuscript; Available in PMC 2025 Oct 10. (PMC11290430; doi:10.1016/j.scitotenv.2024.174379)
Supplement: Supplement1 [file NIHMS2010491-supplement-Supplement1.docx]

Supplemental information for:

**Decay of infectious SARS-CoV-2 and viral RNA in environmental waters and wastewater**

Asja Korajkic^1^*, Brian R. McMinn^1^, Adin C. Pemberton^1^, Julie Kelleher^1^, Warish Ahmed^2^

^1^Office of Research and Development

United States Environmental Protection Agency

26 West Martin Luther King Drive

Cincinnati, OH 45268 United States

^2^ CSIRO Land and Water

Ecosciences Precinct

41 Boggo Road, Qld 4102, Australia

**Table S1.** Average log_10_ reduction values and standard deviation for infectious SARS-CoV-2 and viral RNA at 4℃ in different water types over time.

| Water type | Time | Infectious virus | N1 | N2 |
| --- | --- | --- | --- | --- |
| Primary treated  wastewater | 1h | 0.03±0.29 | -0.19±0.04 | -0.06±0.04 |
|  | 2h | 0.03±0.26 | -0.05±0.05 | 0.05±0.03 |
|  | 4h | 0.48±0.27 | -0.20±0.01 | 0.00±0.06 |
|  | 8h | 0.51±0.58 | -0.24±0.03 | -0.09±0.04 |
|  | 24h | 0.91±0.31 | -0.20±0.03 | -0.01±0.02 |
|  | 48h | 1.17±0.14 | -0.31±0.06 | -0.10±0.01 |
|  | 96h | 1.38±0.22 | -0.19±0.01 | -0.04±0.07 |
|  | 192h | 1.10±0.37 | -0.17±0.02 | 0.09±0.05 |
|  | 264h | 1.57±0.62 | -0.21±0.06 | 0.04±0.08 |
|  | 360h | 1.21±0.37 | -0.18±0.04 | -0.02±0.05 |
| Final disinfected  wastewater | 1h | 0.31±0.09 | -0.21±0.18 | -0.11±0.12 |
|  | 2h | 0.27±0.09 | -0.06±0.18 | 0.08±0.09 |
|  | 4h | 0.67±0.21 | -0.26±0.08 | -0.02±0.08 |
|  | 8h | 0.25±0.12 | -0.32±0.11 | -0.14±0.12 |
|  | 24h | 0.69±0.15 | -0.17±0.10 | 0.03±0.12 |
|  | 48h | 0.89±0.14 | -0.35±0.10 | -0.17±0.09 |
|  | 96h | 0.74±0.12 | -0.27±0.05 | -0.09±0.06 |
|  | 192h | 1.35±0.45 | -0.16±0.07 | 0.10±0.11 |
|  | 264h | 1.06±0.12 | -0.28±0.07 | -0.05±0.06 |
|  | 360h | 0.95±0.15 | -0.15±0.07 | 0.05±0.10 |
| Lake water | 1h | 0.07±0.17 | -0.10±0.09 | -0.03±0.12 |
|  | 2h | 0.19±0.23 | 0.06±0.03 | 0.11±0.06 |
|  | 4h | 0.26±0.05 | -0.03±0.04 | 0.21±0.08 |
|  | 8h | 0.15±0.10 | -0.20±0.02 | -0.07±0.06 |
|  | 24h | 0.84±0.09 | -0.10±0.04 | 0.05±0.07 |
|  | 48h | 1.19±0.12 | -0.25±0.03 | -0.13±0.07 |
|  | 96h | 1.31±1.16 | -0.13±0.05 | -0.03±0.05 |
|  | 192h | 1.48±0.27 | -0.03±0.05 | 0.20±0.01 |
|  | 264h | 2.96±0.66 | -0.18±0.03 | 0.00±0.07 |
|  | 360h | 2.53±0.60 | 0.06±0.04 | 0.22±0.08 |
| Artificial marine water | 1h | 0.17±0.07 | -0.47±0.14 | -0.42±0.16 |
|  | 2h | 0.40±0.13 | -0.06±0.29 | 0.02±0.37 |
|  | 4h | 0.61±0.27 | -0.17±0.40 | 0.03±0.44 |
|  | 8h | 0.27±0.10 | -0.43±0.35 | -0.31±0.42 |
|  | 24h | 0.46±0.17 | -0.01±0.13 | 0.23±0.21 |
|  | 48h | 0.82±0.14 | -0.28±0.06 | -0.08±0.05 |
|  | 96h | 0.68±0.10 | -0.65±0.15 | -0.48±0.15 |
|  | 192h | 0.77±0.67 | -0.42±0.34 | -0.07±0.35 |
|  | 264h | 1.74±0.37 | -0.53±0.11 | -0.36±0.10 |
|  | 360h | 1.45±0.23 | 0.02±0.25 | 0.21±0.30 |

**Table S2.** Average log_10_ reduction values and standard deviation for infectious SARS-CoV-2 and viral RNA at 25℃ in different water types over time.

| Water type | Time | Infectious virus | N1 | N2 |
| --- | --- | --- | --- | --- |
| Primary treated  wastewater | 1h | 0.14±0.44 | -0.12±0.03 | -0.02±0.05 |
|  | 2h | 0.02±0.39 | 0.07±0.16 | 0.19±0.13 |
|  | 4h | 0.28±0.37 | -0.20±0.01 | 0.05±0.06 |
|  | 8h | 0.59±0.27 | -0.38±0.03 | 0.02±0.05 |
|  | 24h | 1.18±0.30 | -0.12±0.06 | 0.07±0.07 |
|  | 48h | 2.33±0.25 | -0.26±0.04 | 0.00±0.02 |
|  | 96h | N/A* | -0.22±0.09 | 0.07±0.07 |
|  | 192h |  | -0.16±0.00 | 0.34±0.03 |
|  | 264h |  | -0.11±0.15 | 0.39±0.18 |
|  | 360h |  | 0.16±0.10 | 0.65±0.08 |
| Final disinfected  wastewater | 1h | 0.39±0.15 | 0.19±0.07 | 0.11±0.05 |
|  | 2h | 0.04±0.11 | 0.35±0.14 | 0.26±0.11 |
|  | 4h | 0.67±0.10 | 0.08±0.13 | 0.15±0.05 |
|  | 8h | 0.36±0.34 | 0.06±0.21 | 0.08±0.10 |
|  | 24h | 1.45±0.19 | 0.15±0.20 | 0.18±0.08 |
|  | 48h | 1.36±0.20 | -0.05±0.12 | 0.01±0.10 |
|  | 96h | 1.46±0.09 | 0.01±0.13 | 0.08±0.06 |
|  | 192h | N/A* | 0.00±0.15 | 0.17±0.09 |
|  | 264h |  | -0.05±0.11 | 0.10±0.07 |
|  | 360h |  | 0.20±0.16 | 0.29±0.11 |
| Lake water | 1h | 0.32±0.23 | 0.09±0.06 | 0.09±0.07 |
|  | 2h | 0.43±0.11 | 0.30±0.15 | 0.27±0.03 |
|  | 4h | 0.95±0.13 | 0.21±0.08 | 0.35±0.04 |
|  | 8h | 0.81±0.22 | 0.03±0.17 | 0.06±0.03 |
|  | 24h | 1.18±0.13 | 0.15±0.12 | 0.20±0.06 |
|  | 48h | 1.59±0.13 | 0.03±0.08 | 0.10±0.04 |
|  | 96h | 1.40±0.18 | 0.07±0.06 | 0.17±0.07 |
|  | 192h | N/A* | 0.08±0.12 | 0.29±0.08 |
|  | 264h |  | 0.01±0.14 | 0.13±0.04 |
|  | 360h |  | 0.16±0.09 | 0.28±0.04 |
| Artificial marine water | 1h | 0.37±0.11 | 0.00±0.34 | 0.02±0.41 |
|  | 2h | 0.59±0.14 | -0.04±0.17 | -0.12±0.25 |
|  | 4h | 0.78±0.15 | -0.17±0.40 | 0.01±0.47 |
|  | 8h | 0.85±0.14 | -0.67±0.33 | -0.61±0.38 |
|  | 24h | 1.23±0.14 | 0.19±0.64 | 0.39±0.67 |
|  | 48h | 1.60±0.09 | -0.56±0.35 | -0.43±0.45 |
|  | 96h | 2.03±0.05 | -0.03±0.28 | 0.13±0.36 |
|  | 192h | N/A* | 0.26±0.24 | 0.40±0.27 |
|  | 264h |  | 0.55±0.39 | 0.78±0.42 |
|  | 360h |  | 0.16±0.32 | 0.36±0.36 |

*No infectious virus was detected at these time points precluding calculation of log_10_ reduction values

**Table S3.** Average log_10_ reduction values and standard deviation for infectious SARS-CoV-2 and viral RNA at 37℃ in different water types over time.

| Water type | Time | Infectious virus | N1 | N2 |
| --- | --- | --- | --- | --- |
| Primary treated  wastewater | 1h | 0.25±0.08 | 0.18±0.03 | 0.15±0.02 |
|  | 2h | 0.40±0.05 | 0.19±0.06 | 0.11±0.04 |
|  | 4h | 0.88±0.13 | 0.07±0.08 | 0.13±0.06 |
|  | 8h | 1.52±0.27 | -0.01±0.05 | 0.05±0.07 |
|  | 24h | N/A* | 0.17±0.03 | 0.42±0.03 |
|  | 48h |  | 0.58±0.04 | 1.00±0.06 |
|  | 96h |  | 1.20±0.05 | 1.60±0.05 |
|  | 192h |  | 1.98±0.06 | 2.33±0.02 |
|  | 264h |  | 2.48±0.11 | 2.97±0.05 |
|  | 360h |  | 2.88±0.17 | 3.39±0.14 |
| Final disinfected  wastewater | 1h | -0.02±0.02 | 0.21±0.00 | 0.14±0.01 |
|  | 2h | 0.05±0.14 | 0.21±0.04 | 0.08±0.07 |
|  | 4h | 0.37±0.08 | -0.01±0.07 | 0.08±0.09 |
|  | 8h | 0.59±0.15 | 0.05±0.11 | 0.01±0.05 |
|  | 24h | N/A* | 0.05±0.08 | 0.16±0.02 |
|  | 48h |  | 0.21±0.14 | 0.36±0.10 |
|  | 96h |  | 0.02±0.06 | 0.21±0.07 |
|  | 192h |  | 0.34±0.05 | 0.67±0.06 |
|  | 264h |  | 0.74±0.36 | 1.13±0.40 |
|  | 360h |  | 1.31±0.28 | 1.74±0.33 |
| Lake water | 1h | -0.16±0.15 | 0.17±0.04 | 0.11±0.01 |
|  | 2h | -0.06±0.19 | 0.17±0.01 | 0.05±0.00 |
|  | 4h | 0.41±0.12 | 0.17±0.01 | 0.29±0.06 |
|  | 8h | 0.66±0.30 | 0.11±0.04 | 0.09±0.07 |
|  | 24h | 0.59±1.03 | 0.22±0.04 | 0.33±0.04 |
|  | 48h | N/A* | 0.39±0.07 | 0.56±0.07 |
|  | 96h |  | 0.32±0.07 | 0.50±0.04 |
|  | 192h |  | 0.61±0.06 | 0.93±0.03 |
|  | 264h |  | 0.87±0.03 | 1.26±0.07 |
|  | 360h |  | 1.43±0.41 | 1.76±0.39 |
| Artificial marine water | 1h | 0.27±0.16 | -0.24±0.17 | -0.33±0.15 |
|  | 2h | 0.40±0.18 | -0.19±0.11 | -0.33±0.16 |
|  | 4h | 0.87±0.20 | -0.38±0.12 | -0.27±0.14 |
|  | 8h | 1.18±0.39 | -0.78±0.18 | -0.86±0.20 |
|  | 24h | N/A* | -0.45±0.17 | -0.27±0.24 |
|  | 48h |  | -0.65±0.15 | -0.49±0.22 |
|  | 96h |  | -0.24±0.32 | 0.05±0.35 |
|  | 192h |  | 0.81±0.16 | 1.21±0.21 |
|  | 264h |  | 0.82±0.18 | 1.31±0.22 |
|  | 360h |  | 1.52±0.24 | 2.05±0.25 |

*No infectious virus was detected at these time points precluding calculation of log_10_ reduction values

**Table S4.** Infectious SARS-CoV-2 concentration data for each time point, matrix and temperature. Standard deviation was calculated for n=3. Initial titer average and standard deviation (n=3): log_10_ 6.259384 ± 0.072133

| Matrix | Temperature | 0h average | 0h stdev | 1h average | 1h stdev | 2h average | 2h stdev | 4h average | 4h stdev |
| --- | --- | --- | --- | --- | --- | --- | --- | --- | --- |
| Primary wastewater | 4℃ | 2.902523 | 0.286738 | 2.875504 | 0.109518 | 2.868277 | 0.0353 | 2.424842 | 0.038652 |
| Final wastewater |  | 3.519921 | 0.063015 | 3.20775 | 0.082161 | 3.24655 | 0.027889 | 2.848023 | 0.150536 |
| Lake Grayson |  | 3.296322 | 0.079849 | 3.22708 | 0.160615 | 3.106715 | 0.15042 | 3.040191 | 0.039604 |
| Marine |  | 3.590683 | 0.022294 | 3.422406 | 0.069268 | 3.189244 | 0.141003 | 2.984748 | 0.278465 |
| Primary wastewater | 25℃ | 2.665212 | 0.325782 | 2.520367 | 0.155509 | 2.648424 | 0.100217 | 2.382868 | 0.107903 |
| Final wastewater |  | 3.478275 | 0.196005 | 3.08976 | 0.041887 | 3.441565 | 0.086996 | 2.807201 | 0.294103 |
| Lake Grayson |  | 3.466443 | 0.11904 | 3.144856 | 0.126138 | 3.040191 | 0.039604 | 2.518768 | 0.241272 |
| Marine |  | 3.59318 | 0.045093 | 3.218444 | 0.067123 | 3.001154 | 0.184051 | 2.815098 | 0.108475 |
| Primary wastewater | 37℃ | 3.119819 | 0.081523 | 2.867353 | 0.153664 | 2.715839 | 0.12311 | 2.236423 | 0.055951 |
| Final wastewater |  | 3.344689 | 0.121736 | 3.360881 | 0.135988 | 3.297736 | 0.065701 | 2.978838 | 0.090586 |
| Lake Grayson |  | 2.893747 | 0.111219 | 3.052787 | 0.045715 | 2.954044 | 0.14148 | 2.483867 | 0.061099 |
| Marine |  | 3.610653 | 0.086531 | 3.335717 | 0.094972 | 3.215141 | 0.094478 | 2.739161 | 0.284275 |
|  |  | 8h average | 8h stdev | 24h average | 24h stdev | 48h average | 48h stdev | 96h average | 96h stdev |
| Primary wastewater | 4℃ | 2.397262 | 0.32694 | 1.99409 | 0.088194 | 1.734707 | 0.153664 | 1.518768 | 0.072133 |
| Final wastewater |  | 3.268364 | 0.059731 | 2.825707 | 0.156048 | 2.631708 | 0.080364 | 2.780491 | 0.055951 |
| Lake Grayson |  | 3.143143 | 0.062523 | 2.46007 | 0.151238 | 2.106715 | 0.15042 | 1.30103 | 0.425721 |
| Marine |  | 3.323759 | 0.082241 | 3.126737 | 0.156048 | 2.770319 | 0.150633 | 2.913072 | 0.098869 |
| Primary wastewater | 25℃ | 2.079181 | 0.261951 | 1.482386 | 0.435074 | 0.333333 | 0.57735 | 0 | 0 |
| Final wastewater |  | 3.118548 | 0.152398 | 2.026394 | 0.045715 | 2.123072 | 0.049096 | 2.019475 | 0.107122 |
| Lake Grayson |  | 2.660757 | 0.101666 | 2.284566 | 0.172005 | 1.881151 | 0.172262 | 2.062507 | 0.075317 |
| Marine |  | 2.739491 | 0.138936 | 2.360881 | 0.097392 | 1.99409 | 0.088194 | 1.560414 | 0.072133 |
| Primary wastewater | 37℃ | 1.60206 | 0.30103 | 0 | 0 | 0 | 0 | 0 | 0 |
| Final wastewater |  | 2.751758 | 0.045715 | 0 | 0 | 0 | 0 | 0 | 0 |
| Lake Grayson |  | 2.234144 | 0.224327 | 1.666667 | 0.055951 | 0.333333 | 0.57735 | 0 | 0 |
| Marine |  | 2.433677 | 0.378688 | 0 | 0 | 0 | 0 | 0 | 0 |
|  |  | 192h average | 192h stdev | 264h average | 264h stdev | 360h average | 360h stdev | |  |
| Primary wastewater | 4℃ | 1.30103 | 0.30103 | 1.333333 | 0.350603 | 1.69306 | 0.088194 |  |  |
| Final wastewater |  | 2.468215 | 0.135909 | 2.455287 | 0.167505 | 2.573772 | 0.087348 |  |  |
| Lake Grayson |  | 1.634363 | 0.055951 |  |  | 1.150515 | 0.21286 |  |  |
| Marine |  | 2.330822 | 0.160159 | 1.852101 | 0.352503 | 2.143143 | 0.238575 |  |  |
| Primary wastewater | 25℃ | 0 | 0 | 0 | 0 | 0 | 0 |  |  |
| Final wastewater |  | 0 | 0 | 0 | 0 | 0 | 0 |  |  |
| Lake Grayson |  | 0 | 0 | 0 | 0 | 0 | 0 |  |  |
| Marine |  | 0 | 0 | 0 | 0 | 0 | 0 |  |  |
| Primary wastewater | 37℃ | 0 | 0 | 0 | 0 | 0 | 0 |  |  |
| Final wastewater |  | 0 | 0 | 0 | 0 | 0 | 0 |  |  |
| Lake Grayson |  | 0 | 0 | 0 | 0 | 0 | 0 |  |  |
| Marine |  | 0 | 0 | 0 | 0 | 0 | 0 |  |  |

**Table S5.** N1 gene copies concentration data for each time point, matrix and temperature. Standard deviation was calculated for n=3.

Initial titer average and standard deviation (n=3): log_10_ 8.68334 ± 0.00123

| Matrix | Temperature | 0h average | 0h stdev | 1h average | 1h stdev | 2h average | 2h stdev | 4h average | 4h stdev |
| --- | --- | --- | --- | --- | --- | --- | --- | --- | --- |
| Primary wastewater | 4℃ | 7.570709 | 0.047626 | 7.763615 | 0.023985 | 7.625037 | 0.023405 | 7.771829 | 0.056224 |
| Final wastewater |  | 7.555526 | 0.066309 | 7.765787 | 0.119629 | 7.610834 | 0.115475 | 7.818049 | 0.036673 |
| Lake Grayson |  | 7.603603 | 0.026827 | 7.704937 | 0.06791 | 7.547802 | 0.026666 | 7.628951 | 0.019157 |
| Marine |  | 6.947543 | 0.052191 | 7.416785 | 0.108161 | 7.002673 | 0.239266 | 7.115367 | 0.359445 |
| Primary wastewater | 25℃ | 7.508825 | 0.054872 | 7.627493 | 0.03969 | 7.438635 | 0.108436 | 7.709164 | 0.058361 |
| Final wastewater |  | 7.801722 | 0.106044 | 7.612953 | 0.060815 | 7.447396 | 0.09291 | 7.720861 | 0.019594 |
| Lake Grayson |  | 7.714515 | 0.102068 | 7.62658 | 0.046187 | 7.417141 | 0.047698 | 7.506504 | 0.029927 |
| Marine |  | 7.213761 | 0.282776 | 7.208977 | 0.326852 | 7.254205 | 0.288681 | 7.381333 | 0.122502 |
| Primary wastewater | 37℃ | 7.694063 | 0.049634 | 7.515204 | 0.019224 | 7.500876 | 0.07646 | 7.626139 | 0.120457 |
| Final wastewater |  | 7.700578 | 0.032441 | 7.490288 | 0.033681 | 7.487153 | 0.059779 | 7.708927 | 0.041849 |
| Lake Grayson |  | 7.63562 | 0.00677 | 7.465603 | 0.042948 | 7.464356 | 0.010824 | 7.462524 | 0.015213 |
| Marine |  | 7.013165 | 0.136869 | 7.252955 | 0.103633 | 7.1992 | 0.188846 | 7.390041 | 0.189285 |
|  |  | 8h average | 8h stdev | 24h average | 24h stdev | 48h average | 48h stdev | 96h average | 96h stdev |
| Primary wastewater | 4℃ | 7.812133 | 0.053472 | 7.765723 | 0.012786 | 7.881448 | 0.010632 | 7.758998 | 0.040774 |
| Final wastewater |  | 7.873076 | 0.081176 | 7.724043 | 0.053878 | 7.909931 | 0.04185 | 7.828548 | 0.024562 |
| Lake Grayson |  | 7.802374 | 0.01648 | 7.7054 | 0.0125 | 7.849989 | 0.036909 | 7.730944 | 0.048781 |
| Marine |  | 7.381644 | 0.380283 | 6.952793 | 0.134321 | 7.228724 | 0.050307 | 7.600523 | 0.161871 |
| Primary wastewater | 25℃ | 7.887091 | 0.078318 | 7.625358 | 0.030145 | 7.764389 | 0.054973 | 7.72729 | 0.031597 |
| Final wastewater |  | 7.739164 | 0.217172 | 7.652208 | 0.094304 | 7.8514 | 0.012771 | 7.79637 | 0.019475 |
| Lake Grayson |  | 7.682599 | 0.06773 | 7.566743 | 0.091776 | 7.688253 | 0.028821 | 7.647779 | 0.054956 |
| Marine |  | 7.883194 | 0.084803 | 7.024879 | 0.424635 | 7.769525 | 0.082676 | 7.241624 | 0.055214 |
| Primary wastewater | 37℃ | 7.708886 | 0.034503 | 7.52575 | 0.032895 | 7.114928 | 0.013546 | 6.498228 | 0.048814 |
| Final wastewater |  | 7.653157 | 0.13783 | 7.653069 | 0.096975 | 7.491562 | 0.139744 | 7.682154 | 0.028528 |
| Lake Grayson |  | 7.521848 | 0.03855 | 7.419355 | 0.038814 | 7.246958 | 0.063105 | 7.311347 | 0.067669 |
| Marine |  | 7.794794 | 0.061222 | 7.459095 | 0.083685 | 7.661904 | 0.076788 | 7.250139 | 0.182107 |
|  |  | 192h average | 192h stdev | 264h average | 264h stdev | 360h average | 360stdev |  |  |
| Primary wastewater | 4℃ | 7.742645 | 0.042104 | 7.780752 | 0.102591 | 7.752999 | 0.010454 |  |  |
| Final wastewater |  | 7.712082 | 0.012055 | 7.83594 | 0.050319 | 7.704362 | 0.006248 |  |  |
| Lake Grayson |  | 7.62967 | 0.02461 | 7.781239 | 0.012225 | 7.539708 | 0.034398 |  |  |
| Marine |  | 7.366655 | 0.299659 | 7.476718 | 0.082633 | 6.928711 | 0.202862 |  |  |
| Primary wastewater | 25℃ | 7.66891 | 0.055464 | 7.615925 | 0.170934 | 7.352096 | 0.040781 |  |  |
| Final wastewater |  | 7.802625 | 0.056496 | 7.856096 | 0.034826 | 7.604937 | 0.056452 |  |  |
| Lake Grayson |  | 7.635223 | 0.055681 | 7.70582 | 0.090643 | 7.553889 | 0.039473 |  |  |
| Marine |  | 6.94947 | 0.178848 | 6.666021 | 0.139618 | 7.048788 | 0.101825 |  |  |
| Primary wastewater | 37℃ | 5.71108 | 0.054206 | 5.215219 | 0.06971 | 4.815181 | 0.128887 |  |  |
| Final wastewater |  | 7.356277 | 0.064039 | 6.958424 | 0.339247 | 6.39322 | 0.279137 |  |  |
| Lake Grayson |  | 7.025748 | 0.064883 | 6.770191 | 0.040106 | 6.203119 | 0.413034 |  |  |
| Marine |  | 6.201112 | 0.149407 | 6.196102 | 0.044338 | 5.496337 | 0.116321 |  |  |

**Table S6.** N2 gene copies concentration data for each time point, matrix and temperature. Standard deviation was calculated for n=3.

Initial titer average and standard deviation (n=3): log_10_ 7.792724 ± 0.01256

| Matrix | Temperature | 0h average | 0h stdev | 1h average | 1h stdev | 2h average | 2h stdev | 4h average | 4h stdev |
| --- | --- | --- | --- | --- | --- | --- | --- | --- | --- |
| Primary wastewater | 4℃ | 7.553789 | 0.008867 | 7.609903 | 0.036788 | 7.500718 | 0.018282 | 7.551332 | 0.050571 |
| Final wastewater |  | 7.560648 | 0.065127 | 7.670316 | 0.066051 | 7.48263 | 0.027459 | 7.578123 | 0.040667 |
| Lake Grayson |  | 7.547781 | 0.06324 | 7.579837 | 0.059061 | 7.435347 | 0.01985 | 7.338944 | 0.01524 |
| Marine |  | 6.885024 | 0.05775 | 7.309166 | 0.156231 | 6.864891 | 0.330351 | 6.853873 | 0.407853 |
| Primary wastewater | 25℃ | 7.544044 | 0.066481 | 7.562194 | 0.036592 | 7.3582 | 0.067315 | 7.493559 | 0.055667 |
| Final wastewater |  | 7.675254 | 0.050488 | 7.564869 | 0.022294 | 7.417625 | 0.061173 | 7.530142 | 0.015624 |
| Lake Grayson |  | 7.611842 | 0.027337 | 7.516905 | 0.053132 | 7.345185 | 0.020414 | 7.26108 | 0.019616 |
| Marine |  | 7.109691 | 0.328744 | 7.090175 | 0.386249 | 7.229831 | 0.292132 | 7.102174 | 0.140009 |
| Primary wastewater | 37℃ | 7.603889 | 0.05018 | 7.452347 | 0.030863 | 7.497594 | 0.067012 | 7.469995 | 0.046178 |
| Final wastewater |  | 7.586863 | 0.040542 | 7.445639 | 0.043877 | 7.509413 | 0.045399 | 7.503235 | 0.046116 |
| Lake Grayson |  | 7.515139 | 0.027304 | 7.40306 | 0.01983 | 7.466814 | 0.029844 | 7.224457 | 0.047078 |
| Marine |  | 6.851185 | 0.171728 | 7.178301 | 0.110967 | 7.185382 | 0.228892 | 7.121464 | 0.235051 |
|  |  | 8h average | 8h stdev | 24h average | 24h stdev | 48h average | 48h stdev | 96h average | 96h stdev |
| Primary wastewater | 4℃ | 7.644352 | 0.042374 | 7.567371 | 0.00958 | 7.655821 | 0.009133 | 7.595821 | 0.058209 |
| Final wastewater |  | 7.704642 | 0.101037 | 7.526668 | 0.055155 | 7.731918 | 0.031863 | 7.647906 | 0.0065 |
| Lake Grayson |  | 7.621318 | 0.010046 | 7.502125 | 0.017372 | 7.681721 | 0.016888 | 7.575831 | 0.027782 |
| Marine |  | 7.193704 | 0.439322 | 6.654844 | 0.172989 | 6.966523 | 0.045996 | 7.36671 | 0.187326 |
| Primary wastewater | 25℃ | 7.52433 | 0.020452 | 7.46967 | 0.007698 | 7.544697 | 0.050867 | 7.469123 | 0.023702 |
| Final wastewater |  | 7.59264 | 0.04965 | 7.49969 | 0.038092 | 7.660885 | 0.046183 | 7.599532 | 0.020206 |
| Lake Grayson |  | 7.552406 | 0.037342 | 7.40856 | 0.053654 | 7.516205 | 0.010382 | 7.445205 | 0.044528 |
| Marine |  | 7.720762 | 0.102993 | 6.720398 | 0.413995 | 7.543206 | 0.135569 | 6.977779 | 0.066171 |
| Primary wastewater | 37℃ | 7.553904 | 0.041377 | 7.185838 | 0.061098 | 6.606261 | 0.008849 | 6.003563 | 0.008286 |
| Final wastewater |  | 7.5748 | 0.073217 | 7.424482 | 0.060264 | 7.223161 | 0.069352 | 7.373857 | 0.038977 |
| Lake Grayson |  | 7.420505 | 0.059159 | 7.185626 | 0.037616 | 6.958354 | 0.064366 | 7.018941 | 0.044367 |
| Marine |  | 7.712158 | 0.075696 | 7.120284 | 0.095285 | 7.343731 | 0.127024 | 6.800098 | 0.1846 |
|  |  | 192h average | a92h stdev | 264h average | 264h stdev | 360h average | 360h stdev | |  |
| Primary wastewater | 4℃ | 7.460065 | 0.040261 | 7.511524 | 0.071296 | 7.573096 | 0.041525 |  |  |
| Final wastewater |  | 7.464085 | 0.050889 | 7.6072 | 0.027556 | 7.510454 | 0.035212 |  |  |
| Lake Grayson |  | 7.345619 | 0.074524 | 7.551073 | 0.033585 | 7.328739 | 0.028673 |  |  |
| Marine |  | 6.95369 | 0.325717 | 7.242453 | 0.080531 | 6.672644 | 0.249208 |  |  |
| Primary wastewater | 25℃ | 7.20825 | 0.054353 | 7.149217 | 0.153388 | 6.891326 | 0.036476 |  |  |
| Final wastewater |  | 7.501575 | 0.047172 | 7.579366 | 0.017138 | 7.385704 | 0.07031 |  |  |
| Lake Grayson |  | 7.32359 | 0.062073 | 7.48015 | 0.052094 | 7.336569 | 0.0518 |  |  |
| Marine |  | 6.709633 | 0.219175 | 6.33433 | 0.146951 | 6.746046 | 0.119083 |  |  |
| Primary wastewater | 37℃ | 5.27033 | 0.039738 | 4.630559 | 0.058807 | 4.211672 | 0.08848 |  |  |
| Final wastewater |  | 6.915673 | 0.040103 | 6.457359 | 0.356452 | 5.849386 | 0.290666 |  |  |
| Lake Grayson |  | 6.583634 | 0.043166 | 6.257328 | 0.098516 | 5.753177 | 0.404211 |  |  |
| Marine |  | 5.638022 | 0.189653 | 5.53659 | 0.044036 | 4.803058 | 0.109921 |  |  |

**Table S7.** Tukey’s multiple comparisons test (effect of temperature). Only statistically significant comparisons are shown.

| **Analyte** | **Time point** | **Water type^a^** | **Average log_10_ reduction and standard deviation** | | **P value** |
| --- | --- | --- | --- | --- | --- |
| Infectious  virus | T_8h_ | P | 4℃: 0.51 ± 0.58 | 37℃: 1.52 ± 0.27 | 0.0084 |
|  |  |  | 25℃: 0.59 ± 0.27 | 37℃: 1.52 ± 0.27 | 0.0189 |
|  |  | M | 4℃: 0.27 ± 0.10 | 37℃: 1.18 ± 0.39 | 0.0234 |
|  | T_48h_ | P | 4℃: 1.17 ± 0.14 | 25℃: 2.33 ± 0.25 | <0.0001 |
|  |  | F | 4℃: 0.89 ± 0.14 | 25℃: 1.36 ± 0.20 | 0.0388 |
|  |  | M | 4℃: 0.82 ± 0.14 | 25℃: 1.60 ± 0.09 | 0.0004 |
| N1 | T_48h_ | P | 4℃: -0.31 ± 0.06 | 37℃: 0.58 ± 0.04 | <0.0001 |
|  |  |  | 25℃: -0.26 ± 0.04 | 37℃: 0.58 ± 0.04 | <0.0001 |
|  |  | F | 4℃: -0.35 ± 0.10 | 37℃: 0.21 ± 0.14 | 0.0013 |
|  |  | L | 4℃: -0.25 ± 0.03 | 37℃: 0.39 ± 0.07 | 0.0003 |
|  | T_360h_ | P | 4℃: -0.18 ± 0.04 | 37℃: 2.88 ± 0.17 | <0.0001 |
|  |  |  | 25℃: 0.16 ± 0.10 | 37℃: 2.88 ± 0.17 | <0.0001 |
|  |  | F | 4℃: -0.15 ± 0.07 | 37℃: 1.31 ± 0.28 | <0.0001 |
|  |  |  | 25℃: 0.20 ± 0.16 | 37℃: 1.31 ± 0.28 | <0.0001 |
|  |  | L | 4℃: 0.06 ± 0.04 | 37℃: 1.43 ± 0.41 | <0.0001 |
|  |  |  | 25℃: 0.16 ± 0.09 | 37℃: 1.43 ± 0.41 | <0.0001 |
|  |  | M | 4℃: 0.02 ± 0.25 | 37℃: 1.52 ± 0.24 | <0.0001 |
|  |  |  | 25℃: 0.16 ± 0.32 | 37℃: 1.52 ± 0.24 | <0.0001 |
| N2 | T_48h_ | P | 4℃: -0.10 ± 0.01 | 37℃: 1.00 ± 0.06 | <0.0001 |
|  |  |  | 25℃: 0.00 ± 0.02 | 37℃: 1.00 ± 0.06 | <0.0001 |
|  |  | F | 4℃: -0.17 ± 0.09 | 37℃: 0.36 ± 0.10 | 0.0137 |
|  |  | L | 4℃: -0.13 ± 0.07 | 25℃: 0.10 ± 0.04 | 0.0007 |
|  | T_360h_ | P | 4℃: -0.02 ± 0.05 | 25℃: 0.00 ± 0.02 | 0.0423 |
|  |  |  | 4℃: -0.02 ± 0.05 | 37℃: 3.39 ± 0.14 | <0.0001 |
|  |  |  | 25℃: 0.65 ± 0.08 | 37℃: 3.39 ± 0.14 | <0.0001 |
|  |  | F | 4℃: 0.05 ± 0.10 | 37℃: 1.74 ± 0.33 | <0.0001 |
|  |  |  | 25℃: 0.29 ± 0.11 | 37℃: 1.74 ± 0.33 | <0.0001 |
|  |  | L | 4℃: 0.22 ± 0.08 | 37℃: 1.76 ± 0.39 | <0.0001 |
|  |  |  | 25℃: 0.28 ± 0.04 | 37℃: 1.76 ± 0.39 | <0.0001 |
|  |  | M | 4℃: 0.21 ± 0.30 | 37℃: 2.05 ± 0.25 | <0.0001 |
|  |  |  | 25℃: 0.36 ± 0.36 | 37℃: 2.05 ± 0.25 | <0.0001 |

^a^P (primary wastewater effluent), F (final wastewater effluent), L (lake water), M (marine water)

**Table S8.** Tukey’s multiple comparisons test (effect of water type). Only statistically significant comparisons are shown.

| **Analyte** | **Time point** | **Temperature** | **Average log_10_ reduction and standard deviation^a^** | | **P value** |
| --- | --- | --- | --- | --- | --- |
| Infectious  virus | T_8h_ | 37℃ | P: 1.52 ± 0.27 | F: 0.59 ± 0.15 | 0.0203 |
|  |  |  | P: 1.52 ± 0.27 | L: 0.66 ± 0.30 | 0.0387 |
|  | T_48h_ | 25℃ | P: 2.33 ± 0.25 | F: 1.36 ± 0.20 | <0.0001 |
|  |  |  | P: 2.33 ± 0.25 | L: 1.59 ± 0.13 | 0.0006 |
|  |  |  | P: 2.33 ± 0.25 | M: 1.60 ± 0.09 | 0.0007 |
| N1 | T_8h_ | 25℃ | F: 0.06 ± 0.21 | M: -0.67 ± 0.33 | 0.0012 |
|  |  |  | L: 0.03 ± 0.17 | M: -0.67 ± 0.33 | 0.002 |
|  |  | 37℃ | F: 0.05 ± 0.11 | M: -0.67 ± 0.33 | 0.0006 |
|  |  |  | L: 0.11 ± 0.04 | M: -0.67 ± 0.33 | <0.0001 |
|  | T_48h_ | 25℃ | F: -0.05 ± 0.12 | M: -0.56 ± 0.35 | 0.0045 |
|  |  |  | L: 0.03 ± 0.08 | M: -0.56 ± 0.35 | 0.0008 |
|  |  | 37℃ | P: 0.58 ± 0.04 | M: -0.65 ± 0.15 | <0.0001 |
|  |  |  | F: 0.21 ± 0.14 | M: -0.65 ± 0.15 | <0.0001 |
|  |  |  | L: 0.39 ± 0.07 | M: -0.65 ± 0.15 | <0.0001 |
|  | T_360h_ | 37℃ | P: 2.88 ± 0.17 | F: 1.31 ± 0.28 | <0.0001 |
|  |  |  | P: 2.88 ± 0.17 | L: 1.43 ± 0.41 | <0.0001 |
|  |  |  | P: 2.88 ± 0.17 | M: 1.52 ± 0.24 | <0.0001 |
| N2 | T_8h_ | 25℃ | P: 0.02 ± 0.05 | M: -0.61 ± 0.38 | 0.013 |
|  |  |  | F: 0.08 ± 0.10 | M: -0.61 ± 0.38 | 0.0048 |
|  |  |  | L: 0.06 ± 0.03 | M: -0.61 ± 0.38 | 0.007 |
|  |  | 37℃ | P: 0.05 ± 0.07 | M: -0.86 ± 0.20 | 0.0001 |
|  | T_48h_ | 25℃ | L: 0.10 ± 0.04 | M: -0.43 ± 0.45 | 0.0152 |
|  |  | 37℃ | P: 1.00 ± 0.06 | F: 0.36 ± 0.10 | 0.0022 |
|  |  |  | P: 1.00 ± 0.06 | M: -0.49 ± 0.22 | <0.0001 |
|  |  |  | F: 0.36 ± 0.10 | M: -0.49 ± 0.22 | <0.0001 |
|  |  |  | L: 0.56 ± 0.07 | M: -0.49 ± 0.22 | <0.0001 |
|  | T_360h_ | 37℃ | P: 3.39 ± 0.14 | F: 1.74 ± 0.33 | <0.0001 |
|  |  |  | P: 3.39 ± 0.14 | L: 1.76 ± 0.39 | <0.0001 |
|  |  |  | P: 3.39 ± 0.14 | M: 2.05 ± 0.25 | <0.0001 |

^a^P (primary wastewater effluent), F (final wastewater effluent), L (lake water), M (marine water)
